# The relationship between parent–child relationship and peer victimization: a multiple mediation model through peer relationship and depression

**DOI:** 10.3389/fnbeh.2023.1170891

**Published:** 2023-07-27

**Authors:** Pingyan Zhou, Jinqi Dong, Jian Liu, Hongbo Wen, Zhe Wang

**Affiliations:** Collaborative Innovation Center of Assessment for Basic Education Quality, Beijing Normal University, Beijing, China

**Keywords:** peer victimization, parent–child relationship, peer relationship, depression, a multiple mediation model

## Abstract

**Introduction:**

Peer victimization is a highly prevalent worldwide issue with cross-cultural characteristics. Parent–child relationship and peer victimization is known to be interrelated, but how they influence each other remains unclear. This study explored the mechanisms of peer victimization related to parent–child relationship.

**Methods:**

A total of 58,756 fourth grade students aged 10–12 years (10.83 ± 0.83, 54.4% males) from China completed four questionnaires. A multiple mediator model was tested, in which the two variables influenced each other through the mediating factors of peer relationship and depression.

**Results:**

Peer victimization was indirectly negatively impacted by parent–child relationship through two chain mediating factors of peer relationship and depression: (1) the mediational path through peer relationship with an effect size of 44.66%; (2) the mediational path through depression with an effect size of 21.64%; and (3) the mediational path through peer relationship and depression with an effect size of 18.08%. The total mediational effect size was 84.11%.

**Conclusion:**

The effect size through peer relationship is the strongest among the three mediation paths, suggesting that peer relationship is the key determinant in breaking the link between parent–child relationship and victimization. Poor parent–child and peer relationships may be risk factors eliciting peer victimization. Compared to internalizing behaviors (e.g., depression), low-quality interpersonal relationships maybe the root cause of the formation and maintenance of victimization. Thus, intervention programs against bullying should pay more attention on children’s contextual factors, especially their relationships with their families and peers, among children at an early age.

## 1. Introduction

For children, being the target of bullying has been the focus of extensive literature over the last three decades (Tsaousis, 2016; Smith and Berkkun, 2020). Approximately 19.9% of children are contemporaneous and longitudinally victimized by their peers (Hong and Espelage, 2012). Olweus stated that peer victimization is a phenomenon that a peer was attacked repeatedly and over time by one or more students with intention (Olweus, 1993). Being victimized is significantly associated with various psychosocial maladjustments, such as feelings of loneliness, school-related fear, anxiety, depression, low self-esteem, and suicide, which can be found in preschoolers (Krygsman and Vaillancourt, 2019), children (Reijntjes et al., 2010; Vaillancourt et al., 2013), adolescents (Van Geel et al., 2014), and even in adults (McDougall and Vaillancourt, 2015). A meta-analysis of 165 studies stated that, compared to their peers, children being victimized display significantly higher levels of depression, suicidal ideation, and suicidal behaviors (Moore et al., 2017). However, the low intervention effects within schools suggest that these programs might have ignored some important factors (Cunningham et al., 2009; Juvonen and Graham, 2014; Lee et al., 2015), thus, it is necessary to explore the key factors (e.g., interpersonal relationships) and mechanisms to improve the appropriateness of intervention strategies.

The parent–child relationship is a unique bond between children and parents or primary caregivers, which impacts children’s physical, emotional, and social development (Bowlby, 1973). Attachment theory postulates that children’s early relationships with parents form an internal working model (IWM) that provides internal representations of self-worth, significant others, and their relationships. Finally, the IWM transforms into an unconscious, automated behavioral pattern that works in the subconscious of children. This is the mechanism by which early relationship quality with parents influences children’s later interactions and relationships with others outside the family (Bowlby, 1973). Multiple studies found that parent–child attachment influenced the quality of children’s peer relationship (Schneider et al., 2001; Gorrese and Ruggieri, 2012; Pallini et al., 2014). For example, mother–child conflicts are positively related to peer rejection and father-child conflicts are negatively associated with peer acceptance (Liu et al., 2020).

Secure attachment predicts healthy adaptation, whereas insecure attachment relationships signal the risks of internalizing and externalizing difficulties, such as peer victimization (Ward et al., 2018; Zhu et al., 2019). Walden et al. found that children with lower attachment levels are more likely to be victims than those with higher levels of attachment (Walden, 2010). Also, studies showed that higher parental rejection and lower parental warmth preceded children’s experiences of victimization (Kaufman et al., 2020). Exposure to negative parenting behavior (e.g., abuse, neglect, and maladaptive parenting) makes children feel powerless and less-confident, and is strongly associated with both victimization and bullying (Baldry and Farrington, 2011; Lereya et al., 2013; Brendgen et al., 2016; Lahav-Kadmiel and Brunstein-Klomek, 2018; Rudolph et al., 2020).

Both negative and positive peer relationships are significantly correlated with victimization experiences. A longitudinal study showed that peer rejection is closely related to and contributes to initiation and sustenance of victimization over time (Hodges and Perry, 1999; Godleski et al., 2015), which positively predicts victimization for fourth graders in a 2-year follow up study (Hanish and Guerra, 2000). Similarly, conflict and betrayal from a close friend increases the odds of victimization (Boulton et al., 1999). In contrast, children’s acceptance by lunch mates predicts decreases in peer victimization (Craig et al., 2016). The reason may be that individuals who interact well with friends, learn social skills, acquire emotional and cognitive supports, and practice interpersonal skills for later relationships, in turn, they can resolve conflict and rejection with adaptive strategies to gain acceptance from friends (Malcolm et al., 2006; Blandon et al., 2010).

The interpersonal risk model hypothesizes that significant pressure related to social relationships engenders problematic outcomes, such as depression (Sentse et al., 2017). Baumeister et al. noted that long lasting peer relational problems cause youth to feel a loss of positive, stable, and continuous belongingness (Baumeister and Leary, 1995), consequently resulting in feelings of depression. Moreover, children with higher family conflicts experience more distress and depression (Goodman et al., 2019), while others with high levels of caring from and connectedness with parents are more likely to report less distress (Jakobsen et al., 2012; Pina-Watson and Castillo, 2015). Longitudinal studies showed that depressive symptoms are predicted by mother–child conflicts for both boys and girls, and by poor father-child relationships only for boys (Branje et al., 2010). Other studies also demonstrated that poor peer relations (e.g., low acceptance, or low friendship stability) preceded depressive symptoms (Prinstein et al., 2005; Lansford et al., 2007; Williford et al., 2012).

Moreover, studies found that depressive symptoms were also important factors related to peer victimization, which were internal characteristics of individuals compared to interpersonal relationships. The symptoms-driven model hypothesized that depressive symptoms are more susceptible to peer difficulties including peer victimization (Kochel et al., 2012; Krygsman and Vaillancourt, 2017; Sentse et al., 2017). For depressed children, depressive symptoms may interfere with the development of adaptive skills associated with initiating and maintaining relationships (Rudolph et al., 2008), which results in many rejections and conflicts in their relationships. Moreover, in communication, depressed children share certain behavioral styles related to vulnerability, such as withdrawal, passivity, and fearfulness (Kennedy et al., 1989; Zalsman et al., 2006), which leads them to be perceived as easy targets to be attacked. A longitudinal study showed that children’s experiences of victimization at age 16 years were predicted by depressive symptoms at age 8 years (Sourander et al., 2000). Similarly, a five-year follow-up study showed that depressive symptoms preceded peer victimization (Saint-Georges and Vaillancourt, 2020) and contributed to self-, peer-, and teacher-reported peer victimization (Kochel et al., 2012; Kochel and Bagwell, 2017).

As described above, it is reasonable to expect that parent–child relationship, peer relationship, depression, and peer victimization are related variables. Peer status had a greater impact than the family environment or internalizing factors on being a victim of bullying (Cook et al., 2010). Although a meta-analysis revealed that the parent–child relationship was a strong predictor of peer victimization (Ward et al., 2018), modest effect sizes reflect that this relationship is likely indirect and mediated by other key variables. Bandura’s triadic reciprocal causation model deemed that a person’s problem behaviors were expected to be closely related to the environment, and their characteristics (such as depression) as well as the interactions between the environment and these characteristics (Bandura, 1978). In addition, the spillover theory posits that one is embedded in multiple interdependent social systems. Changes in social interactions, especially negative aspects, in one system can spill over to other systems through mood, values, skills, and behavior (Edwards and Rothbard, 2000; Kaufman et al., 2020). This study is innovative in that it investigates the effects of contextual factors and internal characteristics on the relationships simultaneously. Thus, we hypothesized that peer victimization was impacted by parent–child relationship through the mediating factors of peer relationship and depression. We formulated the following specific hypotheses: (1) peer relationship mediated the association between parent–child relationship and peer victimization; (2) depression bridged the links between parent–child relationship and peer victimization; and (3) the chain of peer relationship and depression mediated the association between parent–child relationship and peer victimization.

## 2. Methods

### 2.1. Participants

Data were obtained from a cross-sectional school-based survey of fourth grade Chinese students, aged 10–12 years. The survey employed a two-stage (school, student) sample design: (1) all junior schools were selected in Zhengzhou City, China; and (2) within selected schools, all students in fourth grades were involved. Students completed self-administered questionnaires in their classrooms between September and October 2015, which included questions on demographic characteristics, parent–child relationship, peer victimization, peer relationship, and depression. The total sample consisted of 58, 756 fourth graders from 280 urban elementary schools in Zhengzhou City. The official language was Mandarin. Among the children, 54.4% were boys, and 24.4% were singletons. The students were informed by their teachers about the objectives of the survey. Written and oral consents were obtained from all the children, and their parents and teachers, respectively. Children were assured of the strict confidentiality of their responses.

### 2.2. Questionnaire survey

Children answered four questionnaires in this study. All four questionnaires were revised based on the Chinese versions of the translation questionnaires. The revision process was: (1) based on interviews and targeted groups, the items were revised or deleted; (2) the questionnaires were translated back into English to compare the differences between the two versions; and (3) with the pretest data, we analyzed the psychometric indexes of the revised questionnaires. All the indexes conformed to the psychometric criterion. For the Parent–Child Relationship Scale, the pretest Cronbach’s alpha was 0.78. The confirmatory factor analysis (CFA) results showed acceptable fit indices, *χ*^2^_(43)_ = 31,996.12, *p* < 0.001, *χ*^2^/df = 744.10, CFI = 0.87, TLI = 0.83, RMSEA = 0.11. For the Peer Victimization Questionnaire, the pretest Cronbach’s alpha was 0.88. The CFA results showed acceptable fit indices, *χ*^2^_(14)_ = 16,045.20, *p* < 0.001, *χ*^2^/df = 1146.09, CFI = 0.93, TLI = 0.89, RMSEA = 0.13. For the Revised Peer Relationship Scale, the pretest Cronbach’s alpha was 0.82. The results of the CFA showed acceptable fit indices, *χ*^2^_(34)_ = 16,665.98, *p* < 0.001, *χ*^2^/df = 490.18, CFI = 0.92, TLI = 0.90, RMSEA = 0.08. For the Depression Inventory, the pretest Cronbach’s alpha was 0.82. The results of the CFA showed acceptable fit indices, *χ*^2^_(34)_ = 11,183.99, *p* < 0.001, *χ*^2^/df = 319.54, CFI = 0.91, TLI = 0.88, RMSEA = 0.07.

#### 2.2.1. The parent–child relationship scale

To assess the status of the relationship between one and one’s significant others (e.g., parents), the parent–child relationship scale was completed by children. The scale was adapted from the network relationships inventory (Furman and Buhrmester, 1992). The scale has 11 items (e.g., “Are you happy with your parents?”). Participants rated each of the 11 items on a four-point scale (1 = never, 2 = occasionally, 3 = sometimes, 4 = often). The responses were averaged across the 11 items. Higher scores indicated higher PCR quality. The Cronbach’s alpha was 0.775 in this study. The results of the CFA showed acceptable fit indices, *χ*^2^_(43)_ = 31433.69, *p* < 0.001, *χ*^2^/df = 731.02, CFI = 0.86, TLI = 0.82, SRMR = 0.06, RMSEA = 0.11.

#### 2.2.2. The peer victimization questionnaire

Regarding the specific form and frequency of students being bullied at school, children were asked to complete the Peer Victimization Questionnaire, which was revised from the Bully/Victim Questionnaire developed by Olweus (1993). It included seven questions (e.g., “Have you ever been teased or made fun of by other kids in school?”). All seven items were summed to calculate the scale score. Participants independently rated seven items with five choices (0, 1 time, 2 times, 3–4 times, and more than 5 times). Higher scores indicated higher frequency of victimization. The Cronbach’s alpha for this scale was 0.883 in this study. The results of the CFA showed acceptable fit indices, *χ*^2^_(14)_ = 14530.69, *p* < 0.001, *χ*^2^/df = 1037.92, CFI = 0.93, TLI = 0.89, SRMR = 0.04, RMSEA = 0.13.

#### 2.2.3. The revised peer relationship scale

The Peer Relationship Scale consisted of 10 items which was revised from the Children’s Loneliness Scale developed by Asher et al. (1984). Each item was rated using a four-point scale (1 = definitely matches; 4 = definitely does not match). In this scale, peer relationship was reflected by loneliness. Five items required reverse scoring. The Cronbach’s alpha for the scale was 0.823 in this study. The results of the CFA showed acceptable fit indices, *χ*^2^_(34)_ = 15861.62, *p* < 0.001, *χ*^2^/df = 466.52, CFI = 0.92, TLI = 0.90, SRMR = 0.04, RMSEA = 0.09.

#### 2.2.4. The children’s depression inventory: short form

The Children’s Depression Inventory: Short form (CDI: S) was developed by Kovacs (1985). It included 10 items. CDI: S was suitable for children in grades 4–9. Students were required to select one of three choices scored 0, 1, or 2 for each of the 10 items. Higher scores reflected increased severity. A child was classified as having a “depressive tendency” if the scale score was no less than 7. The Cronbach’s alpha for this scale was 0.786 in this study. The results of the CFA showed acceptable fit indices, *χ*^2^_(34)_ = 7579.74, *p* < 0.001, *χ*^2^/df = 222.93, CFI = 0.93, TLI = 0.91, SRMR = 0.04, RMSEA = 0.06.

### 2.3. Data collection and analysis

Statistical analysis was performed with SPSS 18.0 and Mplus 7.0. The significance level was set at *p* < 0.05. Data beyond ±3 standard deviations (*n* = 1,104) and the missing data (*n* = 339) were excluded from the initial cases. Data analyses were performed in three steps. We first performed confirmatory factor analysis (CFA) to test the factor structures, reliabilities, and validities of the four scales. The results of the CFA indicated a good fit to the data for all four scales. Second, we analyzed the association of peer victimization, parent–child relationship, peer relationship, and depression. Third, gender was included as a covariate. Then, a multiple mediation model was performed with Mplus 7.0 to examine the linkages of parent–child relationship, peer relationship and depression to peer victimization, as well as the indirect linkages through peer relationship and depression.

## 3. Results

### 3.1. Bivariate correlations

We calculated the means, standard deviations, and correlations between the five primary variables (Table 1). Parent–child relationship was negatively related to depression and peer victimization, and positively related to peer relationship and gender. Peer victimization was positively related to depression, and negatively related to peer relationship and gender. Depression was negatively associated with peer relationship and gender. Peer relationship was positively related to gender. Independent-sample *t*-tests indicated a significant gender difference (*p* < 0.01) for all four variables (Table 2). Thus, in the following multiple mediation model analysis, gender was treated as a covariate to account for its possible effect on the results.

**Table 1:** Descriptive statistics and correlations between five variables.

Variables	1	2	3	4	5
Gender	1				
Peer victimization	−0.157^**^	1			
Parent–child relationship	0.104^**^	−0.377^**^	1		
Peer relationship	0.130^**^	−0.509^**^	0.541^**^	1	
Depression	−0.076^**^	0.491^**^	−0.540^**^	−0.613^**^	1
Means	1.457	0.930	3.358	3.268	3.197
Standard deviation	0.498	1.00	0.489	0.541	3.095

^**^p < 0.01.

**Table 2:** Independent-sample *t*-test of gender difference.

Variables	Boys		Girls		*t*
	Mean	SD	Mean	SD	
Peer victimization	1.07	1.08	0.76	0.87	38.07^***^
Peer relationship	3.20	0.55	3.35	0.52	−31.51^***^
Depression	3.41	3.17	2.94	2.98	18.23^***^
Parent–child relationship	3.31	0.50	3.41	0.47	−25.12^***^

^***^p < 0.001.

### 3.2. The structural model for testing the mediated effects

**Figure 1:**
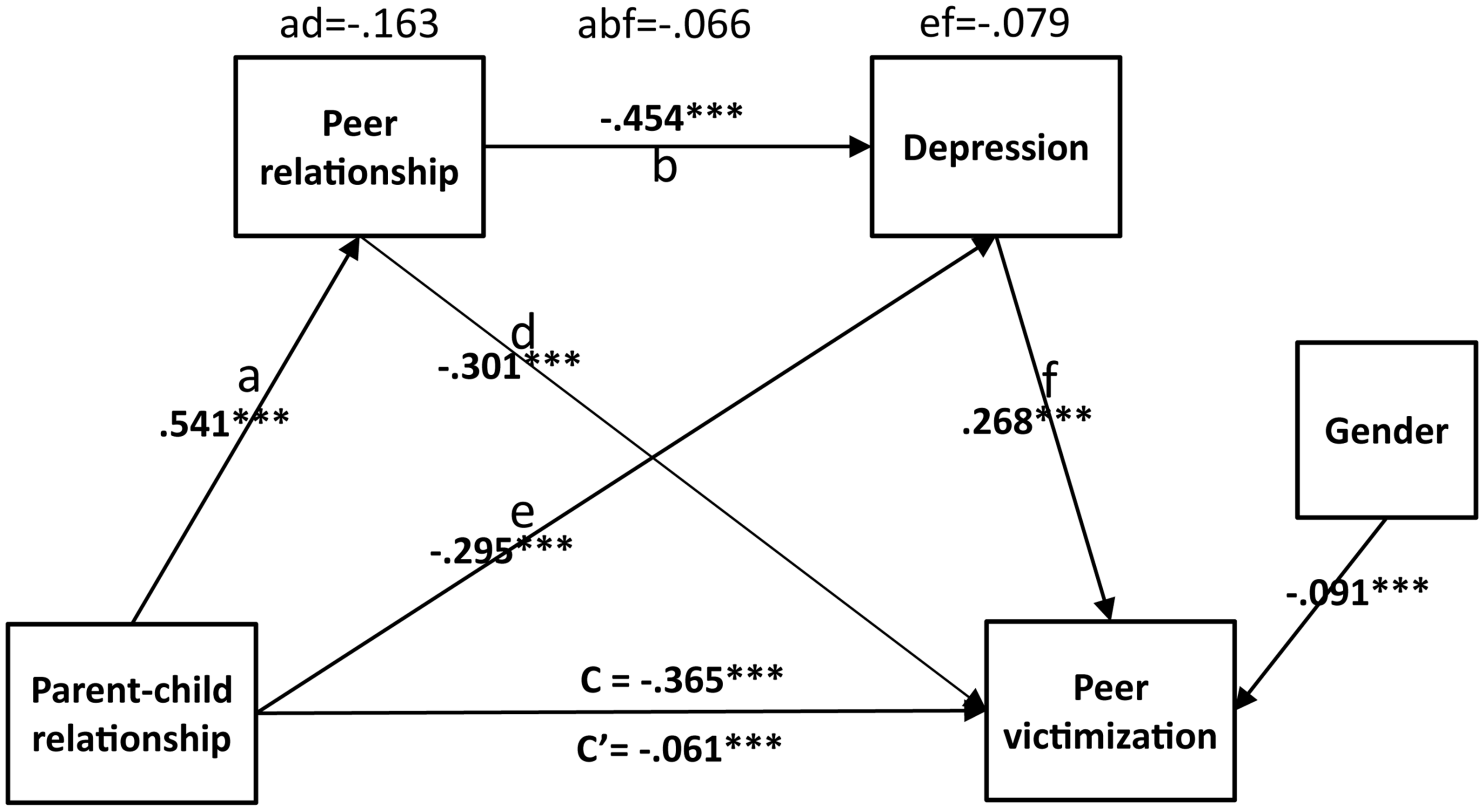
A multiple mediation model of the association between parent–child relationship and peer victimization via peer relationship and depression. Standardized regression coefficients and effect sizes are provided along the paths (^***^p < 0.001). Given the cross-sectional nature of this study, the direction of this model is based on past theoretical and empirical work.

First, regression analysis revealed a significant association between parent–child relationship and peer victimization (ß = −0.365, *p* ≤ 0.001) with gender as covariate variable. Then, by random sampling, 5,000 bootstrapping samples were generated from the original data set (*N* = 57, 313). The multiple mediation model analysis found that the model fits the data well [*χ*^2^_(2)_ = 471.272, *p* < 0.001, CFI = 0.994, TLI = 0.972, RMSEA = 0.064]. The results were presented in Table 3, which showed the indirect effects and their associated 95% confidence intervals. The total effect of parent–child relationship on peer victimization was significant, c = −0.365, *p* ≤ 0.001. After adjusting for the indirect effects of the mediators, the direct effect of parent–child relationship on peer victimization was also significant, but reduced to −0.061, *p* < 0.001. Thus, it was a partial mediation (Figure 1). All indirect effects related to peer relationship and depression were significant as evidenced by confidence intervals that did not contain zero. Thus, peer relationship and depression were all significant mediators. Parent–child relationship was related to peer relationship and depression, and then contributed to peer victimization (Figure 1). The chain mediation of parent–child relationship–peer relationship–depression–peer victimization was significant.

**Table 3:** Bootstrap analyses of the magnitude and statistical significance of indirect effect.

Model pathways	*β* (Estimate)	Effect size	Ratio of total indirect effect	95% CI
Total indirect effect	−0.307	−0.307/−0.365 = 84.11%		−0.313, −0.301
PCR → peer relationship → peer victimization	0.541*−0.301 = −0.163	−0.163/−0.365 = 44.66%	−0.163/−0.307 = 53.09%	−0.168, −0.157
PCR → depression → peer victimization	−0.295*0.268 = −0.079	−0.079/−0.365 = 21.64%	−0.079/−0.307 = 25.73%	−0.083, −0.075
PCR → peer-relationship → depression → peer victimization	0.541*−0.454*0.268 = −0.066	−0.066/−0.365 = 18.08%	−0.066/−0.307 = 21.50%	−0.069, −0.063

Standard errors and test coefficients are values for standardized estimates. PCR: parent–child relationship.

The effect size of the mediational variable was derived from the ratio of the indirect effect to the total effect of the relationship between parent–child relationship and peer victimization (−0.365). The mediating effect of parent–child relationship on peer victimization through peer relationship was −0.163, *p* < 0.001 (Table 3), with an effect size of 44.66%. The mediating effect of parent–child relationship on peer victimization through depression was −0.079, *p* < 0.001, with an effect size of 21.64%. The mediating effect of parent–child relationship on peer victimization through peer relationship and depression was −0.066, *p* < 0.001, with an effect size of 18.08%. The proportion of mediating effects among the total indirect or mediational effect were 53.09%, 25.73%, and 21.50% for the three pathways, respectively. The total mediational effect size of parent–child relationship on peer victimization was 84.11%, which was stronger than the direct effect of parent–child relationship on peer victimization.

## 4. Discussion

Parent–child relationship was significantly related to peer victimization directly, and also indirectly, through peer relationship and depression. This significant direct effect concurs with the existing literature (Baldry and Farrington, 2011; Lereya et al., 2013), which indicates that children with positive (vs. negative) relationships to their parents were less likely to be victimized. When facing victimization, children interacting positively with parents have learned social attitudes and skills from them to maintain relationships with peers (Brendgen et al., 2016) and are also good at adopting adaptive coping strategies to resolve conflict and rejection from peers, which may reduce the risk of further victimization (Lereya et al., 2013). However, the direct effect size was 15.89% vs. the total mediational effect size of 84.11%, indicating that the mediation effect was critical in explaining the relationship between parent–child relationship and peer victimization.

First, we found that peer relationship was a mediator for the association between parent–child relationship and peer victimization among children. Also, 44.66% of the variations in peer victimization related to parent–child relationship were explained by peer relationship, which indicated that poor parent–child relationship triggered peer victimization mainly through peer relationship. According to attachment theory, children’s relationship quality with their parents helps shape their ability to interact with peers (Bowlby, 1973). Children with positive parent–child relationships tend to have higher emotion regulation abilities (Contreras et al., 2000; Baldry and Farrington, 2011) and have learned social skills to cope with stress (e.g., conflict and rejection) in communication, and, thus, they can elicit and maintain friendships (Zimmer-Gembeck et al., 2017). However, those with poor relationships to their parents are not likely to select adaptive coping strategies to tackle conflicts with their peers (Zimmer-Gembeck et al., 2017), and are often immersed in negative moods leading to more hostility and withdrawal behaviors, which in turn the probability of being bullied increases dramatically. Researchers also found that children’s relationship with their parents is closely related to their peer rejection, predicting later experiences of victimization (Liu et al., 2020). The results can also be supported by the spillover theory, which demonstrates that negative relationships with parents abstracted into general knowledge or schema were applied to the peer systems (Edwards and Rothbard, 2000), resulting in peer victimization, indicating that social skills learned from parents for managing conflicts and rejections with peers were the key related to children being bullied.

Second, depression mediated the relationship between parent–child relationship and peer victimization with an effect size of 21.64%. This is consistent with previous studies which suggested that a poor parent–child relationship was a risk factor for the development of depressive symptoms (Jakobsen et al., 2012) and that depression antecedes peer victimization (Kennedy et al., 1989; Zalsman et al., 2006). Children with lower quality relationships to their parents have more depressive experiences and usually have certain social skills deficits (Pina-Watson and Castillo, 2015). They may struggle with reciprocating support and closeness from parents. Additionally, they have negative cognitive beliefs that they are more likely to be rejected by others. To offset their beliefs, they specifically seek more support from their parents, which is believed to induce negative moods in parents. This induces more negative, rejecting responses, and fewer positive behaviors from parents (Hale, 2001), ultimately leading to the onset and maintenance of depressive symptoms for less support in stressful situations (Cobb, 1976). Furthermore, depressive symptoms may induce children to present various vulnerability behaviors, which make them look weak and unsociable, less likely to defend themselves, and unable to retaliate which results in themselves as easy targets for bullies (Kennedy et al., 1989; Zalsman et al., 2006).

This study further revealed that the relationship between parent–child relationship and peer victimization was partially mediated by the chain combination of peer relations and depression, with an effect size of 18.08%. Children with poor parent–child relationship may have more negative peer relationship, which may predict individuals’ depressive experiences, ultimately resulting in more experiences of victimization. Peer victimization is one of the most complicated social and psychological phenomena and is influenced by many personal, behavioral, and environmental factors (Hong and Espelage, 2012; Schacter and Juvonen, 2017). According to Bandura’s triadic reciprocal causation model, children with low-quality relationships to their parents can experience aggravated peer victimization in the context of social and psychological factors, such as poor peer relationship that interacts with depression (Bandura, 1978). Children with poor parental relationship usually have relationship problems with their peers (Zimmer-Gembeck et al., 2017). However, people have a fundamental need for positive and lasting relationships to gain belongingness and esteem (DeWall et al., 2011; Jiang and Liang, 2021). If these children have low peer acceptance and experience more conflicts or rejection, they would suffer more depression and anxiety for the pain of social exclusion (Baskin et al., 2010), finally leading to peer victimization with various vulnerability behaviors.

In conclusion, children with poor parent–child relationship are more vulnerable to peer victimization. Hence, parents should establish warm, sensitive, and supportive relationships with their children since a child was born to deal with peer victimization at its source. Peer relationship is a crucial factor in the intermediary role between the two variables. Looking at all three pathways, the essence of bullying reflects interpersonal deficits, especially in poor peer relationships. Thus, for children, some related abilities must be cultivated to buffer against victimization related to poor parent–child relationship. First, they should constantly improve their social skills and problem solving abilities to maintain positive peer relationship. For example, assertion for boys and connectedness within close relationships for girls should be cultivated (Finnegan et al., 1998). Second, children should improve their emotion regulation capabilities, which could reduce the risk of depression and anxiety related to peer victimization (Adrian et al., 2019). Interventions that incorporate peer relationships aimed at minimizing peer victimization may lessen the negative impact of poor parent–child relationship on children’s social adjustment.

Several limitations to the current study deserve mention. First, all variables were self-reported by participants, which may undermine the validity of the results. Participants may deny experiences of victimization, which may result in unmeasured bias. To reduce this potential bias, future research should collect data from multiple sources (e.g., teachers, parents, and classmates). Second, the current research used a cross-sectional design, therefore a causal inference cannot be drawn. Future longitudinal and/or experimental research can determine the causal relationship among these variables to further verify this model or to explore the bidirectional relationships between victimization and other variables. Third, this research was based on the data collected in 2015. The characteristics of some variables may be changed among 8 years. For example, parent–child relationship may be affected by Two-Child Policy in China. Future research should collect data again to verify this hypothetical model. Finally, the participants are fourth-grade students, which limits the generalizability of our findings. Previous studies shown that younger and older adolescents differ in parent–child relationships (Meeus et al., 2005). For example, at a time of rapid pubertal change, the focus on peers is increasing for adolescents (Liu et al., 2020), thus, the influence of the parent–child relationship on peer relationship may be small, and the corresponding path may change. Future research should separately examine age-related variations and could also investigate whether the hypothetical model of this study holds for particular groups of children (e.g., autistic children, or deaf children).

## Data availability statement

The raw data supporting the conclusions of this article will be made available by the authors, without undue reservation.

## Ethics statement

The studies involving human participants were reviewed and approved by the Institutional Review Board of Beijing Normal University. Written informed consent to participate in this study was provided by the participants’ legal guardian/next of kin.

## Author contributions

PZ designed the study, analyzed the data, and worked on manuscript preparation. JD assisted with data analysis and manuscript preparation. JL was a grant applicant and a project leader and responsible for overall management. HW contributed to data analysis and interpretation. ZW contributed to methodology and data analysis. All authors contributed to the article and approved the submitted version.

## Conflict of interest

The authors declare that the research was conducted in the absence of any commercial or financial relationships that could be construed as a potential conflict of interest.

## Publisher’s note

All claims expressed in this article are solely those of the authors and do not necessarily represent those of their affiliated organizations, or those of the publisher, the editors and the reviewers. Any product that may be evaluated in this article, or claim that may be made by its manufacturer, is not guaranteed or endorsed by the publisher.
